# Involvement of Civil Society Organizations and Other Community Groups in the Response to the Ebola Virus Disease Outbreak in the North Kivu and Ituri Provinces of the Democratic Republic of Congo

**DOI:** 10.29245/2578-3009/2023/S3.1109

**Published:** 2023-05-12

**Authors:** Tieman Diarra, Joseph Okeibunor, Amadou Baïlo DIALLO, Nkechi Onyeneho, Barry Rodrigue, Michel N’da Konan Yao, Zabulon Yoti, Ibrahima Socé FALL

**Affiliations:** 1 Independent Consultant, Mali; 2 World Health Organization; 3 University of Nigeria, Nsukka

**Keywords:** Ebola virus disease, Outbreak, Democratic Republic of Congo, Civil society organization, Community groups, Response

## Abstract

We reviewed the involvement of civil society organizations as well as other community level organizations and structures in the response to the Ebola Virus Disease (EVD) outbreak in the Democratic Republic of Congo. A total of 800 randomly selected adults were surveyed using a uniform set of structured questionnaires. An in-depth interview guide was employed to collect information from community members and religious leaders, while focus group discussions were held with community members. The results revealed some involvement of the different organizations in the communities in the response to the EVD outbreak. However, several challenges were encountered, namely security issues, poor awareness, and non-compliance to safety measures. The findings underscore that despite considerable experience over a long period with outbreaks in the DRC, people still need to be educated about the disease.

## Introduction

The Ebola Virus Disease (EVD) is one of the most dreaded contagious diseases affecting people in the African region. There have been multiple outbreaks of this disease in the Democratic Republic of Congo (DRC). The 10th occurrence in the series of outbreaks was reported in the North Kivu and Ituri provinces. This outbreak, unlike many before it, has been protracted, and the involvement of community organizations and structures has been advocated.

In terms of roles in this effort, community leaders create an activity report and present it to the Risk Communication and Community Engagement team. Community engagement is crucial in the fight against the EVD; Non-Governmental Organizations (NGOs), such as the International Red Cross, have developed an entire process for community engagement in the fight against the EVD in West Africa from 2014 to 2016 (International Federation of Red Cross and Red Crescent Societies, 2017).

Based on collected data, messages were developed considering community needs as well as rumors about the disease. The assessment of the community’s attitudes, knowledge, and beliefs has often been based on a social psychology research approach to foster communication about the disease (Boscarino et al., 2015). Mechanisms are put in place to ensure communication that promotes community involvement. The role of communities is important as they are the main actors in the implementation (Nyarko et al., 2015).

The ability to communicate is crucial, as is the consideration of socio-cultural aspects; these are facilitated by engaging relays who are closer to the communities (Gasquet-Blanchard, 2015). Communication is part of the preparation of communities by the health system (Palagyi, 2019). Knowledge of cultural practices and customs in relation to the EVD is also vital. This includes passing rites and burial practices in general or with specific deceased, such as pregnant women (index case being a pregnant woman).

Information can be tricky to manage and disseminate, as it does not always easily translate into knowledge, even among health professionals (Valerio, 2015). Health system preparation is important (Ngatu, 2016), and capacity building of community members is essential to this preparation. This capacity building has enabled the involvement of communities in health programs such as onchocerciasis control (World Health Organization [WHO], 1996, 1998; Diarra, 1998).

This paper reviews the roles of community organizations and structures, including civil society organizations, in the response to the 10^th^ EVD outbreak in the DRC.

## Study Design and Methods

### Study Design

This study aimed to explore and document experiences and lessons around the response to the 10^th^ EVD outbreak in the North Kivu and Ituri provinces of the DRC. We adopted a cross-sectional design with mixed methods of data collection. The cross-sectional design allows multiple windows of data harvesting, while mixed methods bring the benefits of both quantitative and qualitative approaches and guarantee the integrity and robust interpretation and conclusion that this type of evaluation warrants.

### Selection of study area and population

The study was carried out in the North Kivu and Ituri provinces where the 10^th^ EVD outbreak occurred in the DRC.

**Ituri** is one of the 26 provinces of the DRC. Its capital is the city of Bunia. The Ituri Rainforest is in this area. It is located northeast of the Ituri River and on the western side of Lake Albert. Ituri is a region of high plateau (2000–5000 m) that has a large tropical forest but also the landscape of savannah. The district has rare fauna, including the okapi, the national animal of the Congo. As for flora, an important species is Mangongo, whose leaves are used by the Mbuti to build their homes. The population is composed primarily of Alur, Hema, Lendu, Ngiti, Bira, and Ndo-Okebo, with differing figures on which one of the groups constitutes the largest percentage of the population in the province. The Mbuti, a pygmy ethnic group, reside primarily in the Ituri forest near the Okapi Wildlife Reserve, although some Mbuti have been forced into urban areas by deforestation, over-hunting, and violence. The Kilo-Moto gold mines are partly located in Ituri. In the beginning of the 21st century, petroleum reserves were found by Heritage Oil and Tullow Oil on the shores of Lake Albert.

**North Kivu** (French: *Nord-Kivu*) is a province bordering Lake Kivu in the eastern DRC. Its capital is Goma. North Kivu borders the provinces of Ituri to the north, Tshopo to the northwest, Maniema to the southwest, and South Kivu to the south. To the east, it borders the countries of Uganda and Rwanda. The province consists of three cities—Goma, Butembo, and Beni—and six territories—Beni, Lubero, Masisi, Rutshuru, Nyiragongo, and Walikale. The province is home to the Virunga National Park, a World Heritage Site containing the endangered mountain gorillas. Except for the heightened insecurity and isolation due to rebel activities, North Kivu shares similar demographics with Ituri. The province is politically unstable and since 1998, it has been one of the flashpoints of the military conflicts in the region.

The **2018 or 10**^**th**^ **Kivu Ebola outbreak** began on August 1, 2018, when it was confirmed that four people had tested positive for Ebola virus in the eastern region of Kivu in the DRC (Mahamba, 2018; Mwanamilongo, 2018; Härtl, 2018). The Kivu outbreak included Ituri province, after the first case was confirmed on August 13 (Kuparinen et al., 2018). This outbreak started just days after the end of the 2018 Équateur province DRC Ebola virus outbreak (Weber, 2018; Belluz, 2018)

The affected provinces and general areas are currently undergoing a military conflict, which is hindering treatment and prevention efforts. The WHO’s Deputy Director-General for Emergency Preparedness and Response has described the combination of military conflict and civilian distress as a potential “perfect storm” that could lead to a rapid worsening of the outbreak (WHO, 2019). Due to the deteriorating situation in North Kivu and the surrounding areas, on September 27, the WHO raised the risk assessment at the national and regional level from “high” to “very high” (WHO, 2019).

The study population comprised adults aged ≥ 18 years living in the community as well as response team members. A 2010 estimate reported the population of North Kivu as 5,767,945. A calculation of the population at 70% resulted in a population estimate of 4,614,356. With an annual growth rate of 3.2%, the estimates were reported to be 7,658,406 and 5,360,884 for the general and age ≥ 18 years populations, respectively, in North Kivu. Meanwhile, a 2005 estimate reported the population of Ituri as 4,037,561. An estimate of the population aged ≥ 18 years at 70% resulted in 2,968,865 people. In 2019, the estimates were 6,275,305 and 4,392,714 for the general and age ≥ 18 years populations, respectively.

The response team consisted of over 10,000 persons who were part of different response pillars, including surveillance, risk communication, social anthropology, and vaccination. Others were part of infection prevention and control, treatment and care, safe and dignified burial, security, logistics, and administration, among others.

### Sample size estimation and sampling strategy

#### Sample size

With an assumed 50% chance of accepting Ebola control interventions, at a confidence interval of 95% and with an error margin of 5%, a sample size of 384 was computed for the quantitative study. For the two provinces combined, this resulted in a sample size of 768. This was rounded up to 800 to make allowance for data losses. The sample size of the qualitative study depended on the saturation of information after an initial pair was collected from each category of respondents.

#### Sampling strategy

A multi-stage sampling technique was adopted in selecting the communities, household, and respondents in this study. Two administrative areas (epicenters of the EVD outbreak within each province) were purposively selected. Further, 10 communities were randomly selected from each of the two administrative areas in the province.

### Selection of the households and respondents

The center of the selected community was the reference point where the team spun a pencil to determine the first route and first household; the team thereafter moved to the right to pick the next household and then continued until the number of households to be sampled was included. Where there was a *cul-de-sac*, the step was retraced, and a turn to the left and then to the right was made to continue the sampling process.

Once in a selected household, an adult (≥ 18 years) was randomly selected for inclusion as a participant in the study. The sex of the participants was carefully alternated; if in household #1 a male respondent was selected, in the next household, the focus was on selecting a female respondent.

### Methods

The study was conducted using a mixed methods approach with qualitative and quantitative techniques. The methodology for data gathering included in-depth interviews (IDIs), focus group discussions (FGDs), and surveys using structured questionnaires. This type of study requires a strong focus on individual actors rather than state actors (Babalola & Fatusi, 2009).

Techniques of data collection

**FGDs** were conducted as follows:

A set of questions covering different thematic areas was developed to guide the discussions. The questions covered health care services in the community, awareness of and practices related to the EVD, and assessments of the different pillars of the response interventions.

For the FGDs, 8−12 persons were selected for each session. A minimum of two FGDs were conducted in the selected communities. There were separate FGD sessions for male and female participants in each of the communities. Overall, a total of eight FGD sessions were conducted in each province.

**IDIs** were conducted in each community where FGDs were carried out. The IDIs were held with community/opinion leaders from the selected communities and team leaders of the response pillars. Interviews were used to explore and gain insights on people’s opinions, views, and attitudes related to practices during the outbreak and response efforts, as well as other socio-cultural factors that may have influenced their attitudes toward disease response. The FGD guide was used for the IDI, focusing on the thematic areas of interest to the evaluation.

**A structured questionnaire** was used for collecting quantitative data from households. The questionnaire addressed all the indicators that were used for answering the research questions. The questionnaire was structured with results from the qualitative study. It was categorized into sections: socio-demographic data, perception of health problems in the community, knowledge about the EVD, perceived epidemiology of Ebola in the communities, and sources of information on Ebola. Others included issues on communication and community engagement, infection prevention and control in the communities, vaccination, surveillance, and treatment and care. Other sets of questions covered safe and dignified burial, psychosocial issues, and logistic and security issues.

All interviews and discussions were tape-recorded, and detailed notes were taken simultaneously, including verbal citations. Tape-recorded interviews were transcribed according to standard rules. Observations were also recorded and together with data from the discussions and interviews, these were triangulated with the quantitative data to arrive at conclusions.

### Training and pilot trials

All instruments were ***translated*** into Swahili and French, the common languages spoken in the communities, and back translated to English for clarity of meaning. In each province, ***10 research assistants*** with substantial experience in community interactive research and the use of qualitative and quantitative techniques and cultural sensibility were recruited and trained for three days in Beni and another three days in Bunia on the study objectives and the use of the instrument for data collection. Training also included data entry into Atlas.ti template (qualitative data) and EPI INFO (quantitative data). The instruments were reviewed after training for clarity, understanding, and sensitivity. Each province had a ***supervisor*** who worked with the principal investigator on data quality monitoring, safety advisory, and ethical conduct of the research including the management of informed consent procedures. The study was conducted first in Ituri, then in North Kivu. The lessons learned from Ituri were used to manage the process in North Kivu, a province with more security and logistics challenges. The ***data analyst*** developed and pre-tested the template for data entry and analysis using the pilot test output. Given the short time of the study, data were collected using pencil and paper instead of an android device. Fieldwork was completed in 20 days in each province, before data analysis and report writing.

### Data management

All **quantitative data** were double-checked by the researcher before entering them into a computer. Data were entered into EPI Info and processed using SPSS. Descriptive statistics were used to determine proportions of various categories of respondents and indicators and for comparison. Frequency tables and graphic illustrations were used for presenting the data.

**Qualitative data** derived from the FGDs and IDIs were transcribed from audio records to text. All textual data were analyzed using the Atlas.ti software package. Data were analyzed according to themes corresponding to the indicators in the quantitative data and triangulated during presentation to enable complementary and analogous interpretation.

Given the continuous analytical process involved in qualitative analysis, it is important to note that the initial analysis of the key informant interviews and FGDs informed the final development of the structured questionnaire used in the study. This further enhanced triangulation between the two sets of data collected. While the quantitative results provided us statistical conclusions, the qualitative results placed emphasis on what was said and provided illustrative quotes that added context and depth to the quantitative results.

### Ethical consideration

The principle of do-no-harm was adhered to in the study. Informed study approval was obtained at the levels of the province, local administration, community, and households, while informed consent was obtained from all individuals that were involved in the study. The WHO/AFRO Ethics Review Committee provided ethical approval for the study. All researchers attended the mandatory training, which included substantial discussions on ethical issues in research. With a team constituting 50% female research assistants, same-sex interviews and FGDs were ensured. The assistants were also trained and mandated to comply with child protection and gender sensitivity rules in the process of data collection and visits.

## Results

### Priority health issues in the community

The village chiefs, village groupings, neighborhood chiefs, street chiefs, and avenue chiefs interviewed were among the most accountable people regarding the health problems in their communities. Even in communities that have not been affected by the infection, leaders expressed their concerns about the EVD epidemic. Malaria is the priority health issue in the communities, as it was mentioned by almost all community leaders. Only a few chiefs mentioned headaches, rheumatism, flu, or triploid first. While malaria is always listed as a priority health problem, in several communities, Ebola was not mentioned, especially since some communities had not been affected by the epidemic when this research was being conducted. Diarrhea was mentioned a few times, as was measles. Only one village chief mentioned tobacco and alcohol as health problems in his community, and only two leaders reported poisoning. A chief of Mission village, in the Mandima health zone, said:

As a major health problem affecting our village, we have Malaria, among others; but, for now, what worries us the most is the Ebola… The disease that bothers the population too much is Malaria. Then, it’s typhoid. It is through these two diseases that other diseases find access to the body. As for the Ebola, it is a very dangerous disease. The Ebola kills easily as we have been told through sensitization. Ebola has wreaked a village here. It does more damage than HIV/AIDS.

Village chiefs, heads of village groups, street chiefs, and heads of avenues are involved in the response activities within their communities in all the health areas that were covered by this research, in the North Kivu and Ituri provinces, except for two cases. These were villages that had not yet accepted disease response and were skeptical about the EVD.

### Civil society organizations in the response to the epidemic

Several associations have played an important role in the response to the EVD epidemic. In the North Kivu and Ituri provinces, numerous grassroot community associations were engaged in these activities: youth, women, and professional associations. All communities involved several associations. Some associations were founded less than three years ago, however, most have been active for over a decade. Most of them are self-help associations that survive on membership fees. They provide support to their members if they are affected by an unfortunate event such as an illness or death. Support is also provided for joyful events involving members. Rules are set for the types of support provided; in some cases, the support is in cash. Some associations obtain their resources from the income-generating activities they carry out. Others receive donations or bequests, or rent properties and spaces that belong to them.

All members of the associations we interviewed, in both urban and rural settings, were involved in the response activities to the EVD epidemic. The number of members varied from 10 to dozens. The president of a women’s association in Ngezi, in the Bunia health zone, mentioned:

We raise awareness among the people in the neighborhood. We inform them about the reality of the Ebola because here, many people don’t believe in it. We are doing this even though no one has caught the disease in our neighborhood. We are informing them so that everyone protects themselves against the disease. It has struck elsewhere, and this is a challenge for us. Thanks to our awareness campaigns, people have taken precautions. They don’t greet each other like before.

In the same neighborhood, the president of another women’s association with over 80 members also mentioned similar efforts to raise awareness. In rural areas, associations do similar work. For instance, a men’s association in Katanigwa, a village in the Rwampara health zone, focused on alerting others of the importance of washing their hands with soap.

Furthermore, in Butembo, youth associations are also involved in response activities against the EVD epidemic. A youth association in Beni deals with the training and supervision of young people. It carries out activities to inform and educate members of the community. It consults with local resource persons for the implementation of disease prevention measures in the community. The vice-president of this association mentioned:

We are a structure of supervision, training, and awareness. We inform [people] about the danger of not taking protective measures. It is ignorance that kills. We include the recovered people in the awareness process. We train young people. We have trained more than 300 of them with the agents of the response team. We engage them in response efforts. They do awareness work on a voluntary basis. They are not community relays. Young people include both men and women because we also promote gender equity. We also sensitize in some places where we target bar customers and prostitutes on the Ebola virus disease and on measures to prevent the infection.

Another association responsible for the promotion of gender equity in Beni, *One Girl, One Leader*, was involved in activities to fight the EVD. It also intervened outside the town of Beni, in Goma, Oicha, Mangina, Mavivi, and Mutinga. It is active in the provinces of North Kivu and Ituri. It is an association of girls and women but also welcomes men. This organization engaged 300 girls in the fight against the EVD epidemic. They were trained by the response teams for their participation in implementing preventive measures against the disease. The association also meets with recovered people. During these meetings, people learn about the problems encountered by the recovered people; this helps in finding solutions to the struggles related to their social reintegration. The association worked with other organizations involved in the response to the EVD epidemic.

Some associations have diversified their activities in the context of the response to the Ebola epidemic. This is the case of a women’s society that operates in 13 districts of the town, in Beni. They essentially work with women and provide them with information. The vice-president of this organization said:

We sensitize the women dressmakers, saleswomen, and farmers. But we also sensitize other members of the community. We conduct sensitization with a document that we received from the people of the response team. We raise awareness by going from door to door. We organize talks, we use certain radios, we go to churches and schools. We conduct video forums, which are an opportunity to interact with the population on the subjects discussed. We talk about rumors to prevent them from being a source of reluctance. We have helped reduce resistance among women. Most did not believe in the disease. I have personally been to the ETC. I have also met people who have been cured. Recovered people give testimonies at our awareness meetings.

In Beni, another youth association for the development of their neighborhood, Kalinda, is active throughout the town and in other communities in the Beni territory. The association is involved in providing information on community deaths and alerting people to cases of disease. It contributes to raising awareness on preventive measures.

The head of Avenue Ngezi in Butembo emphasized the role of the youth in the fight against the EVD. He mentioned:

Above all, the WHO must also include our avenue youth in the outreach team. Even with a megaphone, they can be of great help to those who cannot move anymore, especially older adults, so they are informed of what is happening in their community, in their surroundings.

Furthermore, the members of the associations we interviewed have received training on the implementation of preventive measures. The vice-president of an association in Beni spoke of the importance of training. He said:

The training strengthened our capacities. During our outreach activities, we were always asked questions. We couldn’t cope because we didn’t have the answers to all the questions that the participants in our outreach meetings were asking us. After the training, we are now able to answer the questions we are asked. But there are still questions that have nothing to do with the epidemic. We have to say that we cannot answer them. Outside our association, members of the Community Action Committee (CAC) have been trained. The heads of neighborhoods, the heads of cells, the heads of 10 houses have also been trained. If a visitor arrives at someone’s house, he informs the head of the 10 houses who then informs the people of the response. We were informed of this provision during the training.

However, not all the members of the associations we met have been vaccinated against the EVD.

The heads of the associations spoke of the difficulties they face in their work. The first difficulty is rumors—some community members talk about resources coming from the response team and consider the disease as a pretext. There are many rumors, according to most of the association members we have interviewed. The vice-president of the mutual mentioned that some community members think that the associations make money by reporting deaths. Another difficulty is the suspicion regarding corruption among association members by the response personnel. The vice-president of an association in Beni mentioned: “*They talk about us as mε nza: that has swallowed something*.” Next, criticism and threats were reported as challenges by a member of an association: *“All those who talk about Ebola, after Ebola, we will find them and deal with them.”* Another challenge is hostility from community members.

One member of an association spoke about the projectiles often fired at them during activities in the community. Additionally, association members lack the means to travel to work in the communities. Insufficient means or media for communication is another difficulty; some people spoke about the lack of megaphones and the lack of support or framework to report their activities in the context of the response to the EVD epidemic. Furthermore, there is a lack of credits for communication. Some association members talked about the high number of phone calls they make during their activities. For them, support to cover such expenses would be of great help. Moreover, the resistance of some members of the community is an obstacle to their activities. Finally, the refusal to follow certain advice, such as to go to the health center or to the hospital in case of an illness, can be a major challenge.

The members of the associations brought proposals to improve the response efforts. These include: 1) greater involvement of the associations; 2) strengthening the capacity of the associations through training; 3) support from partners for the involvement of associations; 4) provision of more hand-washing kits to the communities; 5) adoption of preventive measures by community members; 6) systematic hand washing, especially in places of burial; 7) supervision of the recovered people and support for their families; 8) engagement of young people in all communities; 9) involvement of community leaders; 10) changing the response teams every six months to avoid routine or change the location of the teams; 11) involvement of the hierarchy at the community level—village chiefs and neighborhood chiefs; 12) involvement of local people, especially in awareness-raising activities—some people spoke of the importance of involving indigenous people who, in most cases, are better known by the communities than the response staff, and some mentioned that community members trust people they know more than those they have never seen before; 13) involvement of local organizations, such as NGOs; 14) involvement of response personnel who are respectful toward communities; 15) provision of door to door information, especially for members who do not own a radio; 16) use of fewer vehicles in community activities—some members of the associations mentioned that vehicles are scary, especially when they come in large numbers; 17) use of video forums to inform and raise awareness, and the involvement of family members in safe and dignified burial (SDB); and 18) organizing visits to the ETC to further educate the community members on the reality of the EVD.

**Figure 1:**
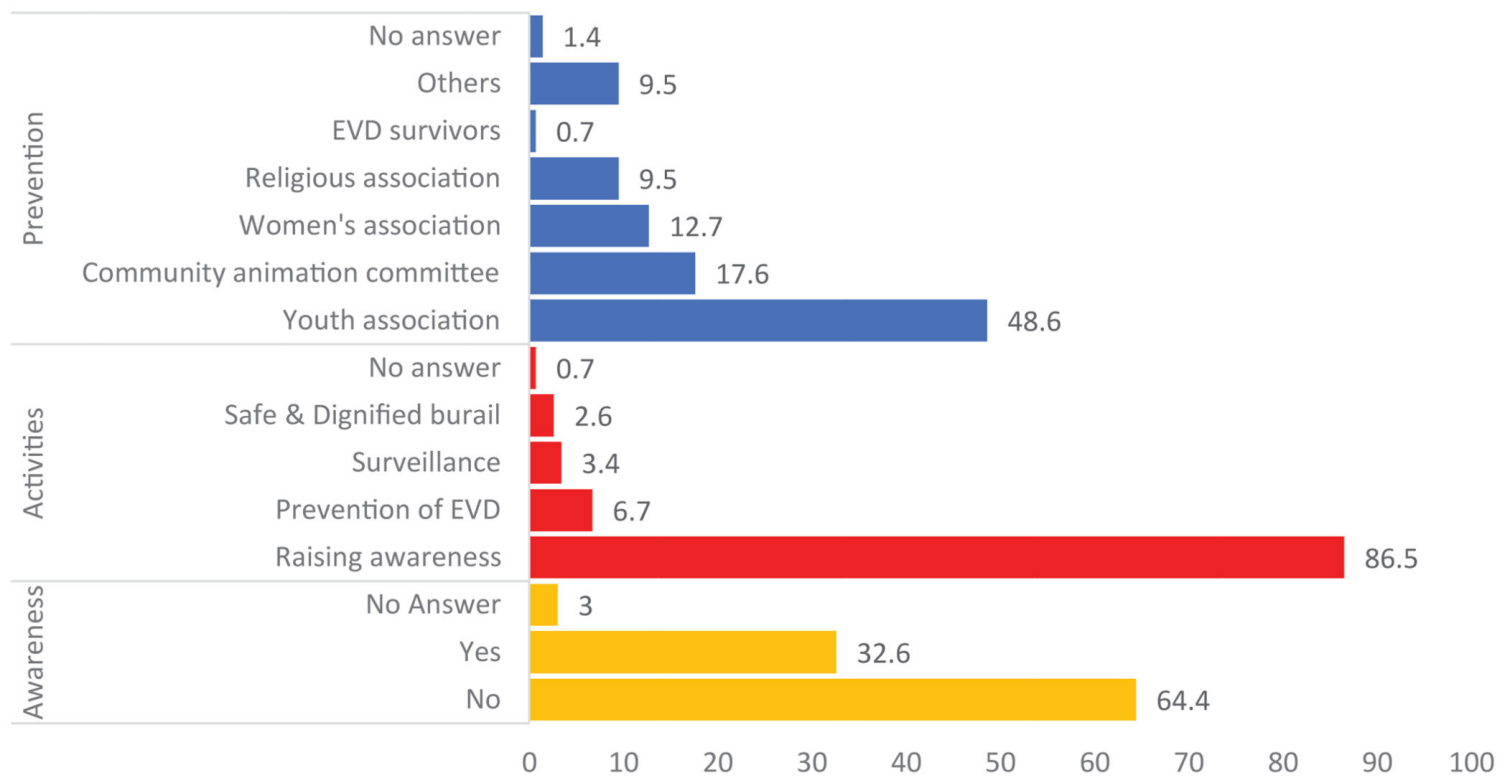
Respondents’ perceptions on the involvement of community structures and organizations in the response to the EVD threat in the DRC

Apart from the interviews, the issue concerning the involvement of the associations in the fight against the EVD epidemic was explored through a survey involving the community members. Figure 1 shows the respondents’ awareness of community association members’ involvement in activities to help prevent EVD.

Less than one-third of the respondents were aware of community associations involved in the EVD response activities. In most cases, community associations are involved in raising awareness among the inhabitants of the village or neighborhood. They are also involved in activities for prevention and control, followed by community-based surveillance and SDB. The associations involved are diverse. Youth associations are found to be the most frequently mentioned, followed by the community animation committee. Women’s associations and religious associations are cited, as well as associations of those who have recovered from the EVD. Over a dozen other community associations were mentioned, including self-help associations, mutual benefit societies, and professional associations.

**Figure 2:**
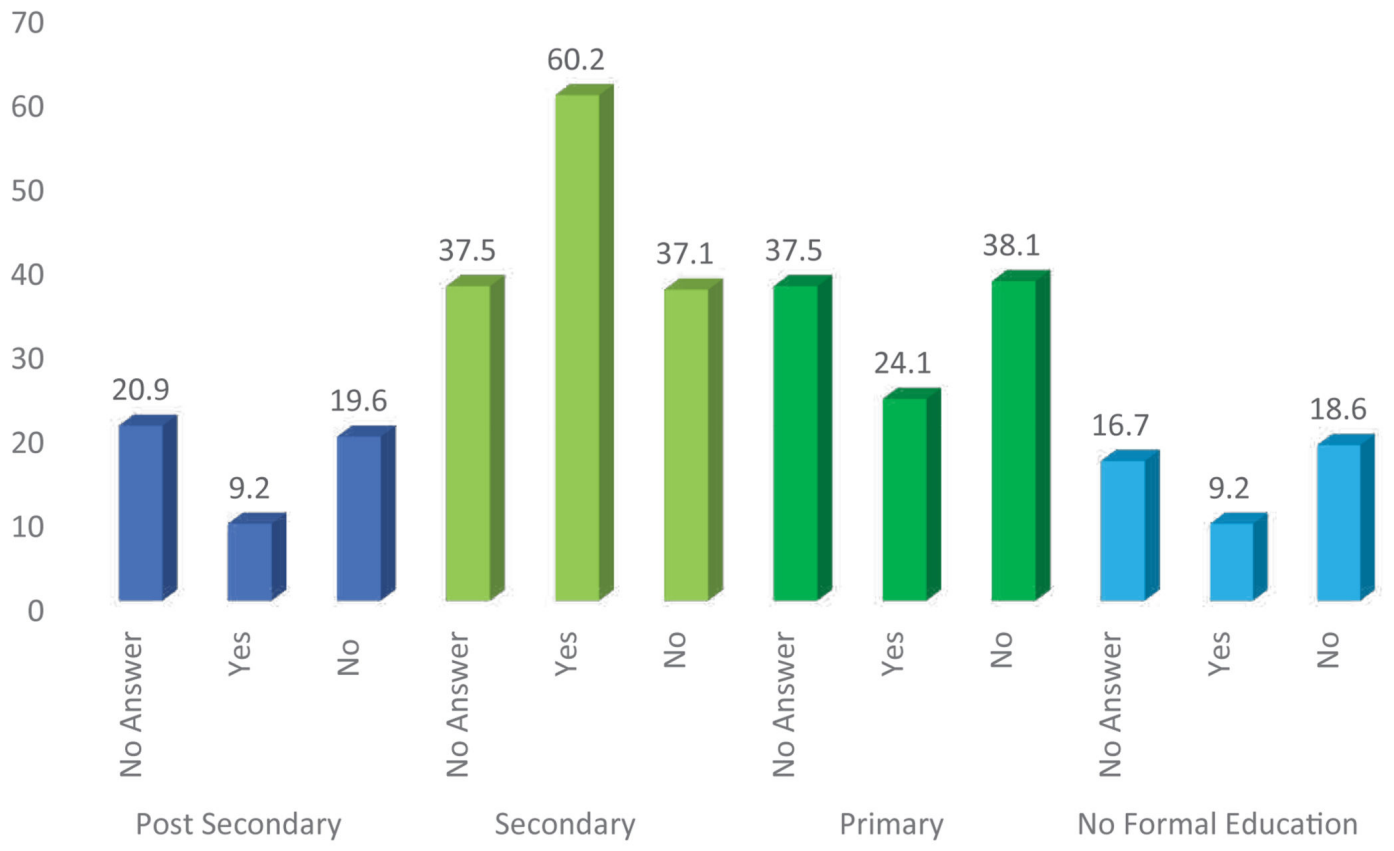
Respondents’ awareness on the involvement of community groups in the EVD response activities, based on their levels of education

Those with a secondary-level education were found to be more likely to believe that community associations are involved in the EVD control activities (Figure 2). This, however, may be influenced by the weight of these people in the entire sample. Nevertheless, this was reported by 43.97% people who had attained a secondary-level education and only 23.50% people who had attained a primary-level education. Moreover, 37.77% people who had attained a graduate-level education stated that community associations are involved in the fight against the EVD epidemic. The difference is significant with a χ^2^ of 34.634. It appears that people with a secondary level of education were not better informed about the activities of the fight against the EVD epidemic.

### Village chiefs, village groupings, neighborhood chiefs, street chiefs, and avenue chiefs

Village, neighborhood, avenue, and street chiefs are important to any activity that takes place in their communities. Therefore, they have been involved at various levels in the response to the EVD epidemic in the North Kivu and Ituri provinces. The chief of the Ngezi district spoke about the role of the district chiefs. Talking about the protection of community members, he said:

Us chiefs are the first to protect community members. That is why, prior to their arrival, members of the response teams must get in touch with us chiefs. Because from us, they can access the people, and we will be able to ensure their protection.

He mentioned that any movement toward an avenue must be reported to the chiefs of this avenue beforehand, so security measures can be taken before the team approaches the population. He stressed the importance of going through the community leaders. He reported that the population is respectful of its chiefs, which is also the case in his district, Ngezi. These words indicate the importance of the involvement of village, neighborhood, avenue, and street chiefs in the implementation of activities in the response to the EVD epidemic. Village chiefs, once informed of health problems, inform the members of their communities in turn. Moreover, they welcome the members of the response teams before they begin their work. They provide their consent to conducting response activities before the arrival of the response teams, as stated later by a village chief.

### Involvement in the response to the EVD outbreak

Village chiefs and leaders of village groupings are involved in the Rwampara health zone, not far from Bunia. Some leaders of village groupings have been working in the same position for more than 20 years. This is the case of one chief in Gblogu. Village chiefs, neighborhood chiefs, street chiefs, and avenue chiefs are involved in several activities.

A leader of a village grouping in the Rwampara health area spoke about his sensitization work:

I called the chiefs of the villages for which I am responsible. They came; I sensitized them on preventive measures, including the importance of hand washing and cleanliness. I hold meetings in schools, churches, and drinking places. In turn, village leaders do the same work when they return home. There are people who don’t believe in the existence of the EVD. They say it is a rather evil disease. They think it’s witchcraft.

The village chiefs work with the healthcare personnel who assist them with awareness-raising activities. Village and neighborhood chiefs are involved in community-based surveillance. The communication and community engagement commission of the Beni coordination works with chiefs and holds regular meetings. These chiefs report on their activities within the community in the context of community-based surveillance and even community watch. In another coordination unit in Beni, Butembo, a similar system is in place. The head of the district of Boikene told us about the work carried out in his district within the framework of community-based surveillance:

To identify cases as quickly as possible, as soon as there is a case of disease, we send an alert. This is a very effective way of getting involved. Through the alerts, the people of the response team usually arrive with the ambulance to take the patient.

The chief of the Mabesele neighborhood, in Oicha, also spoke of his actions. He reported the need to identify the sick as soon as possible and inform the nurses. Moreover, according to him: “*Here, we do a lot of monitoring of the people. As soon as we call, an ambulance arrives, and the people of the response team do their job properly*.” In some cases, the patient is directly sent to the nearest health center to be examined. The chief of the Matembo district, in Boikene, mentioned that this is only possible because information is given to community members beforehand, involving everyone. For him, this is particularly important as some community members do not believe in the existence of the EVD. The chief of Mambango Avenue, in Boikene, informed us about their activities in the context of community-based surveillance:

In the avenue, we make the rounds every time (twothree times a day). This allows us to detect cases if there are any. In addition, the heads of 10 houses send us information in real time, but often, the response agents come late! That’s not right! They must correct this attitude to avoid the spread of the disease.

Village, neighborhood, avenue, and street leaders are involved in case-finding activities in their communities. Village, neighborhood, avenue, and street chiefs are also involved in community watch. In the Beni coordination unit, it is an integral element of community involvement. The heads of villages, neighborhoods, avenues, and streets serve as relays to the heads of families; thus, they can monitor the arrival of people in their households or in their plots. However, this strategy is not formulated in the same terms everywhere. The chiefs were trained as part of the response to the EVD outbreak. The training covered several aspects. The chief of a group of 12 villages in the Rwampara health zone spoke about his training:

I was trained at the Bunia General Hospital. The training lasted three days. It brought together village chiefs, heads of neighborhoods, and the mayor of the town. Other workers from the Bunia town hall also participated.

The chief of the Manjombo village, in the Mandima health zone, explained his training during a workshop: *“It was a three-day training workshop with over 100 people. So, in this area, most of the population is informed.”* In the same area, the chief of the Pygmy camp village described the content of the training he attended:

During our training, we were told that, when a person dies of Ebola, even if it is your brother, you should not go near his body or touch him but leave the burial to the personnel qualified for that, such as the members of the Red Cross. They can bring a family member to check if the deceased really is their dead brother.

The chief of the Boikene district told us how he applies the learning he acquired during his training: *“According to me and the training we received; we all have the obligation to protect ourselves. This is possible with the application of different means of prevention.”* Some village leaders also work in collaboration with the health facilities in their communities. Some are members of the health committee of their community’s health facility, and some are even chairpersons of the CAC to which villagers are associated. This involvement in health activities helps promote the awareness of village, neighborhood, street, and avenue chiefs within the communities.

The village chiefs and neighborhood leaders are involved in informing the members of their community. This is the case for the chief of the Mambango district, in Boikene, who said:

As a community leader, I do everything I can to educate those who have little information or who are poorly informed. I provide them the real information. I do everything to manage the bad information so that the community is not exposed to the danger of the disease.

The village, neighborhood, avenue, and street leaders also contribute to the training of other community members for their involvement in the response activities. The chief of Avenue Baye, in Butembo, told us about his actions in the region:

We are training community relays and they are the ones who go from house to house to inform and raise awareness based on the training they received. This is already beginning to pay off because, as the relays explain to the community members that the disease exists and that it has harmful consequences, they tell them how to protect themselves against this disease. The health centers are helping us popularize the messages. This work has an impact as sensitization continues, and we change information strategies whenever it is necessary.

### Involvement of village chiefs in managing insecurity

Some village chiefs were waiting to be rewarded for their involvement in the management of insecurity. Others do not understand the involvement of the state’s defense and security forces in the *response* efforts. This is the case of the head of the district in Boikene:

We, in the community, wonder if the Congolese army has become a doctor to treat people? The Ebola has come to transform everything. The policemen, our soldiers, have abandoned their task to go only to the WHO and let the criminals take advantage of it to cause us harm. They are still integrating all the authorities in the area, including the customary authorities, to protect the bad acts that they cause during the execution of their work, which nobody accuses.

Community members have often attacked the defense and security forces in a context of lack of information on their role in the response efforts. However, in some cases, village chiefs intervened to put an end to such actions. This is the case of a neighborhood chief who understands their role; he said: *“I facilitate the free access of the police and the military in my village in their activities in my neighborhood. I prevent people in my community from threatening them*.” Earlier, in this neighborhood, there were security incidents that included the destruction of the treatment center, threats to the SDB team members in the Mambango cemetery, and destruction of response vehicles by community members. According to him, these incidents have led to the interruption of the response activities in the neighborhood. However, he mentioned that these security incidents are due to “*the behavior of the agents of the response*.” Moreover, he reported that the recruitment of local workers has helped calm the population. For him, this has greatly contributed to the management of security incidents.

Owing to the insecurity of community members, response teams could not always visit the villages. According to some village chiefs, this insecurity was also due to the population’s lack of confidence in the work of the response teams and the national authorities.

The equipment of the response agents has often been ransacked as some villages have remained hostile to the EVD response and all activities related to the disease. This is the case of some villages in the Mandima health zone. Thus, when a village chief was asked about the apparatus set up in his community, he replied: *“We don’t have any equipment at home, and we don’t want it.”* He also expressed skepticism about the *response* and commented on why the outbreak has persisted: *“It’s because the people involved in this Ebola thing are not serious. They tell too many lies in their favor just to depreciate our community members.”* In some villages, there are no *response* initiatives, and the community members must bear the uncertainty. This is the case in another village in the Mandima area; the village chief said:

In our village, we have destroyed all these materials, and we are chasing away all the response teams. Moreover, you are lucky to be greeted by us because you lowered yourselves to our level of humiliation in front of us. If it wasn’t for that, you would have left a long time ago. By the way, hurry up and leave our village before we become upset. Yes, here we chased out the response team and broke their equipment. You should start by getting informed before coming to us. This is another world; this is not Congo. We are telling you that we do not joke around here. The military or police have no access here. Because the government wants to play with our souls. They told lies in our name. These people have fled, you too should quickly get out of here and not come back. Otherwise, you will experience the same as your predecessors. Go and ask them what happened to them here.

Incidents also occurred elsewhere in the town of Beni. A neighborhood chief spoke of the population’s reaction to a ministerial delegation: *“Projectiles were fired at the minister’s delegation here. The young people did it. Besides, it was the armed group that made the gravediggers’ team flee the cemetery.”* However, the neighborhood chief was involved in managing the incidents; he mentioned:

We managed the incidents by sensitizing the population. But also, it is said that the child who is not taught or educated by their parents will be taught by the world. And as there are many deaths, people begin to understand that death is at our door. The community members understood that it is a disease because, after some died, others still got infected. So, slowly, people have started to understand.

The chief of Avenue Baye, in Butembo, believed that some conflicts with the population arise from the practices of the response. According to him, community members who are recruited and lose their jobs do not help to calm the situation. They become enemies of the response team and try to demonize them by saying, for example:

These people are lying to us; the Ebola does not really exist. They are creating this disease to destroy you, who are doing this work. They will tell you the salary increases with the deaths. This really creates conflicts and adversities in the response.

There has been an evolution in the attitude of community members thanks to the sensitization. The chief of Avenue Baye, in Butembo, shared:

For the members of the response team who travelled by car, there was a problem. At the beginning, if people saw a response vehicle, it was an opportunity to attack it. They managed to injure those inside, not even sparing the driver. When people were informed that the team would come to bury someone, the neighborhood would get mobilized to attack them and manage to open the body bag to see if the person was inside. This led to the spread of the disease. This happened here in Ngesse, as in the commune of Vulengera. As for the destruction of the health center, when someone was told that a person had died there, the angry population came to destroy it. This was the case with the Buchundo center. The incident was very unfortunate for agents of the response team.

Some village chiefs shared the reasons behind these incidents, according to them. For some, it was due to the population’s lack of information. Some community members believed in the rumors that claimed the funding of health units and centers would increase with the number of deaths. For others, the SDB team’s vehicles were attacked because community members wanted to bury the dead themselves. However, the chief of Avenue Baye, in Butembo, mentioned another reason:

If people were attacking the response teams, it was because they not only wanted to bury their relatives themselves but also because they wanted to check whether the person inside the coffin really was their relative. People doubted that the deceased was their relative. Sometimes they thought that the bodies that did not belong to them were given to the families.

The head of the avenue organized daily meetings with members of his community, especially those belonging to associations. The meetings are held in churches and in schools which, according to him, are the best places to disseminate information on the disease and on the activities of the *response* team.

During meetings held by village, neighborhood, avenue, and street chiefs, information is provided on the reality of the disease and the unfoundedness of rumors, as well as those on the competence of members of the response teams. The deconstruction of rumors is important to facilitate a positive involvement of community members (Niabalamou, 2015). Further, the neighborhood chief of Ngezi, in Bunia, spoke of negative reactions from community members due to lack of information. He mentioned a case of the arrival of a *response* team:

Here in our health center in Ngezi, a community member had lost his child, and alerted the response team. When the response team arrived, the population did not know what they were doing. People thought they had come to take the body. But we intervened by calming the population and we let the members know that this team had just come for a test. Then, everything went well.

For the chief of the Madududu village, the lack of information is at the origin of hostility toward community relays and members of the SDB teams who often face verbal threats. However, owing to his interventions, it has never led to violence against those involved in response efforts. He also talks to community members about the importance of the action taken by the Congolese government to end the epidemic. Moreover, he asks members of the response teams to not be discouraged by signs of hostility toward them.

### Involvement of community leaders and resource persons

In addition to village, neighborhood, avenue, and street chiefs, other community leaders have also been involved in the response to the EVD outbreak in both North Kivu and Ituri provinces. Community leaders and resource persons include the opinion leader and teachers of the village or neighborhood. Wise men have been identified and involved in the response activities. One participant, from the Ngezi district, in Bunia, said that wise men have been identified in all districts in addition to the chiefs, and that they have been prepared to be involved in response efforts. He informed us about the training:

We have all been trained. It was about the disease, the symptoms, prevention measures, and the activities we must carry out in our neighborhood. These activities include sensitization, providing information on people arriving at our place, especially those coming from Beni, Butembo, and Mambasa. If we learn of new cases of illness in the community, we must also report them, as well as any case of death that occurs in the neighborhood. If there is a corpse, the response team comes to test if the person died of Ebola or not. Based on our training, we sensitize the community members. We focus on cleanliness, hand washing. We conduct sensitization during the community meetings that we organize. But we also use the radio for this purpose. We have been recruited as wise voices. We are heard in our neighborhoods. We sensitize others. We raise awareness against rumors.

In the Mabasele district of Oicha, a community leader spoke about the actions he is taking against the reluctance of some members. He said:

We inform community members about the activities and practices to prevent the Ebola infection, such as hand washing, avoiding contact, especially with someone who starts to show symptoms, to refer this person to the hospital…People listen to us. Here, some people do not wash their hands because everyone has their own behavior. We know it and still we insist on hand washing. When we see that a member of the community is misinformed, we take care of him. We also provide information in the churches. We organize meetings in the neighborhoods. This work has already been successful.

A community leader from the Mbimbi district, in the Mabasele area, also spoke about his work as a leader:

I sensitize the population. I pass on information in the context of prevention of the EVD. The avenue chiefs and the district chief support me throughout my activities. For those who do not know about things well, who are not well informed about the disease, we go to the field to tell the population to avoid politics in the fight against the disease. Some people make it a political issue. We are giving more information about the Ebola. We are giving community members the toll-free number to call and alert the response people if they need it. We tell community members and relatives of the patients not to touch them anymore. We ask them to call the people of the response team to take care of them, to test if he or she has Ebola or not.

A female community leader in the Bunia health zone, also emphasized the importance of involving community leaders in the response to the Ebola outbreak. She spoke about the fact that many members of her community think that the EVD is an imaginary disease ***malali ya mabe***. She sensitized her community members and made suggestions for the authorities in charge of the response efforts in her locality to put certain measures in place. She mentioned:

I would have liked to see a compulsory hand-washing barrier in my community. Because our village is the entrance to the city of Bunia. I am too afraid that people will pass through here, go to Bunia without being checked and come back. I am scared. We are exposed to this disease. I hope that the government and its partners will act in this regard.

The Chief of Avenue of Ngezi emphasized the role of community leaders as an interface between the population and the members of the response teams. For him, they must also ensure the protection of the members of the *response* teams. He said:

We, the leaders, are the first protection of the response team. Therefore, before their arrival, they must contact us, the chiefs. Because from us, they can get through to the populations. And we will be able to ensure their protection.

He mentioned a case where they intervened to allow the *response* team to perform their work. They prevented negative reactions from community members and especially attacks against the response teams, as stated before.

Village, neighborhood, avenue, and street chiefs and community leaders made proposals for an improved response, as did the leader of OPAS, the village chief. These include equipping the community with more hand-washing facilities; increasing the number of protective equipment in neighborhoods, avenues, and in front of churches, mosques, and other places of worship; securing the response agents; integrating the local population in the response activities; and constructing a health center.

### Religious leaders’ involvement in response efforts

Religious leaders are also involved in response efforts in many ways. While some are sensitizing their followers on the EVD and the means of prevention, others are denying the disease and questioning the reality of the response. We conducted interviews with religious people of both categories. Only some do not get involved in these activities; we only met two such people. Some religious people have other roles in the community. They are also headmasters of schools and are thus involved in the fight against the EVD as well as in their places of worship. Most religious leaders we interviewed have been living in their communities for over three years, and others, for over 14 years. They have been practicing these occupations for a long time—we met a pastor who has been active for 55 years. These religious people ensure the effective use of prevention measures in their places of worship: churches, parishes, and mosques.

While most religious leaders we interviewed had received training to be involved in response activities, particularly for awareness-raising, some had not. This was the case of a pastor in Avenue Ngesi, in Bunia, and another in Katanigwa, in the Rwampara health zone. However, all of them disseminated information about the EVD. A pastor in Ngezi said: “*I have not received any training. I give information about the Ebola to the congregation from what I hear on the radio*.” He has no collaboration with health personnel but is in contact with healthcare workers who pray with him. He has not been vaccinated and did not know there was a vaccine for the EVD. This was also the case of a religious leader at K11, in the Mandima health area. He was an exception as all the other religious leaders we met had been vaccinated.

While some have provided facilities for hand washing in their places of worship, others have not. This is the case of the cleric of the Katanigwa village, not far from Rwampara. As he believes the disease is not present in his community, he did not consider it necessary to take this measure. Moreover, according to him, the practices of the faithful have not changed regarding the observance of measures to prevent the disease in the place of worship. According to him, everyone washes their hands at home before coming to the parish.

From the interviews with pastors, it appears that some passages of the Holy Bible can be used in the context of sensitization to the EVD. The pastor of PK 11 mentioned some of them: in the first book, in the case of Elisha falling ill with a fatal disease, he appealed to the king. This pastor had not been vaccinated. His wife, who is a nurse, is involved in the fight against the EVD outbreak in Mambassa. We interviewed a pastor from a neighborhood in Beni who was not involved in response efforts. He was trained on the EVD as a magistrate. The meeting was mainly motivated by the need to advocate for his involvement in the response in the same sense as the response authorities. We had information about the messages he was giving to the faithful in his place of worship. They were focused on denying the reality of the EVD. He asked us what the reasons for this interview were, as he found it suspicious. We explained that he was not the only religious person we met during this research. He asked us to cut the interview short as he felt he was very busy. We had been able to address the key issue: the support of religious people in the EVD response.

We discussed the issue of the EVD based on its being presented by some as a political set-up. He talked about the challenges due to rumors in families. He talked about his participation in a radio program in Kiswahili and in Kinande. He said he could find a topic that is like the EVD, which could be an opportunity to talk about the disease. He has been hosting this show since 2015. He mentioned that there are families who have left Beni because of Ebola. He made proposals to improve what is being done in the context of response efforts. According to him:

We must always listen. We must not give up because there are fewer cases now. There are people who have left who can return. It must be said that it is not over. Hand washing has dropped. There is a total decrease in vigilance.

Another cleric had been trained twice on the EVD and its means of prevention. He is also the headmaster of a school in Rwampara. He spoke about the involvement of all community leaders in the response to the epidemic. He said that the Holy Bible can be used to raise awareness about the EVD.

According to him, hygiene has its place in the Holy Bible. He referred to the exodus and spoke of God’s orders to Moses requiring latrines for sanitation reasons. He also talked about verses that speak of sickness and healing in Matthew, Luke, and John. According to him, all these verses speak about caring for the sick. Luke was a physician as well as an evangelist.

Attention was also paid to the body in the Holy Bible, which can be useful, according to some religious leaders. For them, it indicated that SDB is not an attack on the body. Great attention is given to the body of the deceased in the communities; he explained:

In the Gospel according to John in the epistles to Paul, in the Old Testament, we speak of the burial. The same is true in Matthew and Luke. The Acts of the Apostles in the Old Testament can be referred to.

His church was fully involved in the response to the EVD outbreak. He made proposals for better success in response efforts. He also mentioned that even the most remote churches must be involved; churches must be equipped with hand-washing facilities; teachers should be trained and involved in the fight against the epidemic; the population should be vaccinated; and eradication of the EVD should be emphasized.

Muslim religious leaders are also involved in response efforts. This was the case of one of the mosques in Butembo where they worked for four months with the WHO. According to the Imam of the first mosque in Butembo, they were told that there has been a change, and they are no longer involved. They were involved in sensitization. According to him, hand washing is part of Islamic rituals. They have reinforced this practice. The Imam also talked about verses in the Quran that can be used in the fight against the EVD. He talked about chapter 5 of the Quran, verses 5 and 6. He said that hygiene has a big place in the Quran and in the practice of Islam. He talked about five things from the Quran in particular: health, memory, wealth, personality, and dignity. Anything that harms health is forbidden and is *haram*, he says. He also mentioned chapter 2 with verses 155 and 214. According to him, there are verses to console the families in case of death. Chapter 20 also has verses that can be used, and he talked about verse 55.

He was trained along with approximately 20 other Muslims from the three mosques in Bembo. However, he was not vaccinated.

He made the following recommendations: the population should not oppose the response; we should help the politicians fight back; we should let competent people handle the response to the epidemic; we should allow all religious people to be involved; we should allow people from all religions to be associated: Muslims and Christians; and we should comply with the EVD prevention guidelines.

A parish vicar in Butembo supports the fight and believes that the EVD must be dealt with: *“It is not a story of* ***wafumu***, *witchcraft, or whatever, no. But the tragedy is that to be treated, one must be totally separated from their relatives and put in the hands of specialists.”* The church was involved in Butembo and asked the faithful to implement all the advice that the medical staff provides to avoid this disease and fight it. The church members have been attending conferences on the EVD periodically and have also been learning about the disease. The religious leaders are involved in response efforts because the church is involved in initiatives that support community health. The parish vicar of Butembo spoke about the health facilities that come under the church such as the hospital in Kyondo, built by religious women, the companions of Mary; he had not been vaccinated. He recommended the following: continuing to talk about this disease and how it spreads; getting people to accept and act according to the advice given to them; informing people about the EVD, and how to prevent it; ensuring that the staff involved are healthcare workers, because afterwards, it is the people who are welcomed who suffer, according to him; providing people access to a permanently toll-free number; raising awareness of pressure groups, particularly among the young; and accepting the existence of the disease.

### Contacts in the response efforts

During the epidemic, some people were contacts of the patients. They received medical follow-up. Interviews were conducted with more than 12 such contacts. As of November 1, 2019, 5,940 contacts were followed-up. Of them, 5,058 had been seen in the last 24 h, a follow-up rate of over 85%. People became contacts through a family member infected with the disease, a neighbor, a friend, or colleagues. A female contact in Makasi, in the Butembo area, said:

I did not become a contact person through my family, nor through my professional activity, but rather because of visiting a friend. I went to visit her in a health facility. Three days later, my friend called to inform me that he had treated a person who had tested positive on the day of my visit. As a precautionary measure, my friend, the nurse, suggested that I agree to be followed up because you never know, one might have used the same chair of the patient.

Some people experienced high levels of anxiety about being a contact and had a difficult time coping with this situation. Some stated that they thought more about death than about life. Others said they were frustrated by the behavior of the members of the team responsible for their follow-up. Many mentioned problems they faced during the follow-up period, including the following. Some contacts felt rejected by family members. One female contact said, *“I was almost rejected, and all my brothers and sisters stayed at a distance from me, so they almost wouldn’t approach me.”* The economic activities of some contacts were compromised. The same female contact said:

Although I own my restaurant, which helps me earn some money, I have seen the number of clients decrease significantly. They have fled since that contact period and have not come back even though the contact period is over. I am also a victim of stigmatization from my fellow entrepreneurs. They refuse to touch the money I give them in exchange for small bills to give to some clients who come to get food. All this happened because the follow-up contact officers came to inform about my situation while many of my clients were eating at the open-air restaurant and this affected me. To this day, only customers who do not know that I have been a contact are eating at my restaurant. My subscribers have changed location. So, I feel uncomfortable. The neighbors of my restaurant, as well as those of my home, are a bit indifferent toward me.

Stigma affected the contacts’ relationships with other members of the community. Some contacts also spoke of the stigma, the shame they felt, and the isolation they experienced from their parents and especially from their children and spouses. Others spoke of the anxiety of contracting the disease and the stress they experienced every day before the follow-up team came. However, for many, the stigma ended after a while. Being a contact has contributed to the recognition of the reality of the EVD.

Overall, contacts were satisfied with their follow-up period and especially with the work of the follow-up workers. One female contact mentioned:

In my contact experience, I was really satisfied by the fact that the people who did not know me took their time to follow up on me, to call me, to come regularly to take my temperature, and that too every day. Really, let them go ahead, we don’t want the disease to persist here at home, our daily activities are already affected.

A contact from the Boikene neighborhood, in Beni, also spoke of his anguish upon learning that he had become a contact. He experienced his contact status as guilt. He mentioned: *“My difficulties were resolved especially after the 21 days expired. I was cleared and started a normal life with the other members in the community.”* The visits and advice of community relays were appreciated by the contacts. The contacts still did not appreciate the fact that the follow-up team addressed them in public often during the first visit. They would have liked to be contacted in confidence.

Some contacts spoke of the importance of their involvement in the response efforts. A woman from the Katia village, near Butembo, said:

We have had a lot of information about the Ebola. We must now help our relatives, inform them so that they go to the health center in case of illness. We are talking to neighbors now to go to the hospital or health center in case of illness. We are asking to inform of cases of death.

They made proposals to improve the work carried out in the response to the EVD outbreak, including: ensuring confidentiality during the first meeting; taking precautions by calling contacts in the context of insecurity; informing the community about the importance of reporting any cases of illness; informing the competent authorities of the presence of any foreign person whom one may be hosting; providing free medicines for people who visit health facilities; accepting the response teams; providing adequate water supply—some spoke about the lack of water in their village, neighborhood, or town; strengthening the control at the barriers even though there are fewer cases of the EVD now; providing protective and preventive devices to combat the disease; supporting children who have been orphaned by the disease; supporting women who have been widowed by the disease; and following up with the recovered people.

### The community in the response efforts

All the people we interviewed had information on the EVD epidemic, even if they live in a community that has not been affected. They were aware of the preventive measures. They also had information and advice about the disease. For instance, a man from Beni described the disease as follows: *“It is a serious disease. It is a politicized disease because it appeared at the time when the elections were going to take place.”* Some people had heard of Ebola before the outbreak in the provinces of North Kivu and Ituri, but most only heard of it because of this outbreak.

A resident of the Ngezi district, in Bunia, reported that he believes in the existence of the EVD, not because he has heard of it but because a member of his family fell ill and was cured after being treated at the ETC. The people we interviewed do not usually talk about the EVD with their neighbors or friends. They talk about it with friends, at home, at school, at work, and in their places of worship. However, others do not. They also had information about response activities. A woman from the same neighborhood said:

I have not seen a single Ebola patient, but I have learned a lot. I know that if someone is sick with Ebola, the people around them are followed by medical staff for a while. I was told that but haven’t seen it.

From the interviews with the various people we met, it appears that they were aware of the preventive measures against the disease. They were also aware of rumors about the infection. While they all believed in the existence of the disease, they all knew members of their community who did not believe in it as well as people who refused the vaccine. One contact mentioned reasons why some refused the vaccine: *“People have many reasons for refusing the vaccine. Some don’t believe in the disease; others say there are two kinds of vaccines. It is the fear of getting the wrong shot that makes them refuse the vaccination.”*

Mostly, the public we interviewed were not vaccinated. However, some people think that only the people involved in the response efforts are vaccinated. They made proposals to improve the response efforts, including involvement of young people in awareness-raising activities; vaccination of all community members; and application of preventive measures.

### Challenges in the fight against the epidemic and community perspectives

**Figure 3:**
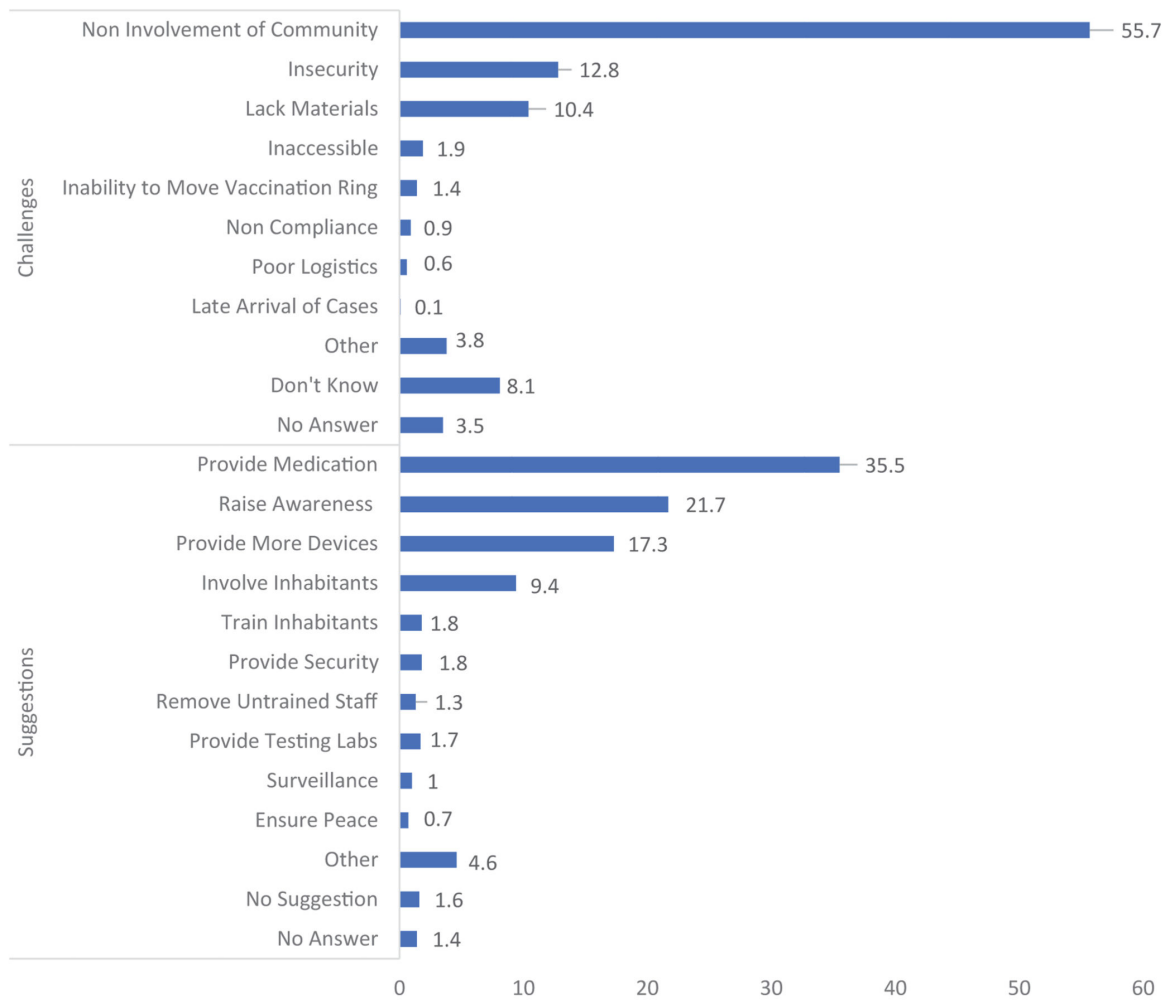
Distribution of respondents by perceived challenges and suggestions.

Apart from the public, the community members also made proposals for the successful fight against the EVD epidemic, based on their experiences and viewpoints. The population surveyed by questionnaire spoke about what they consider to be the greatest difficulty in the fight against the epidemic, as shown in Figure 3.

The reluctance of some village or neighborhood members to participate in the response activities was the greatest difficulty reported by more than a quarter of the participants. The lack of involvement of community members was reported as the second greatest difficulty, followed by security. We have discussed the involvement of community members, but this involvement was considered a problem by many respondents. Indeed, the reluctance of community members was also highlighted.

Among the people interviewed, treatment was found to be the priority. They wanted access to medicines to treat the EVD. More than a quarter of the respondents asked for community members to be made aware of the disease. They also asked for more prevention devices within the community, followed by the involvement of the inhabitants of the village, of the neighborhood and, therefore, of the members of the community. The first three proposals were made by 80% of the respondents. The integration of all health centers in the response efforts was also requested, followed by the training of the inhabitants of the village or neighborhood.

## Discussion and Conclusion

The Risk Communication and Community Engagement team in Beni has hired community leaders who work with members of the team to develop an agenda of activities. Community leaders are responsible for implementing these activities. They meet regularly with the Risk Communication and Community Engagement team professionals to review the implementation of planned activities and strategize for the future.

Based on the information that community members receive from their leaders, they observed prevention measures. The community leaders work with the village, neighborhood, avenue, and street chiefs and, in some cases, with the traditional chiefs. Sometimes, they hold these positions. They participate in community-based surveillance. They call the nurses or team members of the response. When there is a death from the EVD, community leaders negotiate with family members, as is reported by a community leader in Bembo. Some young people also become community leaders and play a major role in the EVD response activities. Youth who are community leaders are heard by the people; they also intervene in cases of reluctance to certain activities such as DSB. People’s reluctance has been an obstacle in the response to the epidemic and this reluctance has often resulted in hostility toward health personnel (Zerbo & Amadou, 2015). This is a barrier to community involvement. Other problems such as the lack of materials were also reported.

People involved in implementing preventive measures had been trained or briefed to enable them to perform their work. The various coordinating bodies of the response teams ensured the capacity building of these people. This capacity building for community involvement started with the training of community engagement staff. The Risk Communication and Community Engagement team provided training for the team members who work with communities. Training has been offered in all response activities conducted during the EVD epidemic. In Sierra Leone, the involvement of community members through sensitization and social mobilization has been to improve their level of information and to adopt practices conducive to the control of the epidemic (Bedson et al., 2020). An assessment of knowledge, attitudes, and practices helps guide the capacity building of community members (Bedson et al., 2020).

Communities have played an important role in the response to the Ebola outbreak in the provinces of North Kivu and Ituri. Several community members have been involved in the response efforts. These include community leaders, such as village, neighborhood, street, and avenue chiefs, and Catholic and Muslim religious leaders. They also include religious people, teachers, and traditional healers, who were involved due to their role in the community. Contacts of people infected were also involved in the response to the epidemic. Moreover, the public, such as patients’ relatives and community members, were involved in these activities. Civil society organizations have also played an important role in the response. They have worked in both urban and rural areas.

Community members have been sensitized and informed about the response activities, especially about the prevention of the disease. They were informed through the radio, media, or social networks, or by neighbors. Posters were also used to provide information. Community leaders organized meetings to inform members of their community. Community involvement was facilitated by the capacity building of specific members by staff involved in the response activities. Thus, they have been identified and trained in their responsibilities as community health workers or community relays. Members of civil society associations have also been trained.

Community members were also involved in awareness raising, community-based surveillance, active case finding, contact tracing and, in some cases, community watch. Community leaders played a specific role in managing insecurity; they identified the EVD as a priority health issue and were committed to fighting the disease. Community members often took actions against the response teams that ranged from reluctance to violence against the teams. They saw security as a critical issue in the fight against the EVD epidemic. Difficulties in community involvement include the reluctance of some village or neighborhood members to participate in the response activities. This has been an obstacle to the effective participation of community members. However, above all, insecurity, which was rather commonly prevalent among community members, has been the greatest obstacle in the response to the EVD epidemic.

## Figures and Tables

**Table 1:** Distribution of participants in the IDI and FGD sessions by provinces

Target	North Kivu Province				Ituri Province			
	Butembo		Beni		Mbuti		Bunia	
	IDI	FGD	IDI	FGD	IDI	FGD	IDI	FGD
Pillar leads	All		All		All		All	
Pillar members	2/pillar		2/pillar		2/pillar		2/pillar	
Community leaders^1^	≥2/ community		≥2/ community		≥2/ community		≥2/ community	
Leader of survivor group	≥2/ community		≥2/ community		≥2/ community		≥2/ community	
Community adults: men		≥2 groups		≥2 groups		≥2 groups		≥2 groups
Community adults: women		≥2 groups		≥2 groups		≥2 groups		≥2 groups
Community male youth		≥2 groups		≥2 groups		≥2 groups		≥2 groups
Community female youth		≥2 groups		≥2 groups		≥2 groups		≥2 groups
Survivors		≥2 groups		≥2 groups		≥2 groups		≥2 groups

1 Community leaders, here, included traditional, religious, political, social opinion leaders and influencers

## Data Availability

The data that support the findings of this study are not publicly available as they contain information that could compromise the privacy of the research participants. The data are available from the corresponding author (Joseph Okeibunor), upon reasonable request.
